# Marginal metallic state at a fractional filling of ’8/5’ and ’4/3’ of Landau levels in the GaAs/AlGaAs 2D electron system

**DOI:** 10.1038/s41598-021-94563-0

**Published:** 2021-07-22

**Authors:** R. G. Mani, U. K. Wijewardena, T. R. Nanayakkara, Annika Kriisa, C. Reichl, W. Wegscheider

**Affiliations:** 1 Dept. of Physics and Astronomy, Georgia State University, Atlanta, GA 30303 USA; 2 Department of Physics, ETH Zurich, 8093 Zurich, Switzerland

**Keywords:** Condensed-matter physics, Quantum Hall, Electronic devices

## Abstract

A metallic state with a vanishing activation gap, at a filling factor $\nu = 8/5$ in the untilted specimen with $n= 2 \times 10^{11} cm^{-2}$, and at $\nu = 4/3$ at $n=1.2 \times 10^{11} cm^{-2}$ under a $\theta = 66^{0}$ tilted magnetic field, is examined through a microwave photo-excited transport study of the GaAs/AlGaAs 2 dimensional electron system (2DES). The results presented here suggest, remarkably, that at the possible degeneracy point of states with different spin polarization, where the 8/5 or 4/3 FQHE vanish, there occurs a peculiar marginal metallic state that differs qualitatively from a quantum Hall insulating state and the usual quantum Hall metallic state. Such a marginal metallic state occurs most prominently at $\nu =8/5$, and at $\nu =4/3$ under tilt as mentioned above, over the interval $1 \le \nu \le 2$, that also includes the $\nu = 3/2$ state, which appears perceptibly gapped in the first instance.

## Introduction

Two-dimensional electron systems subjected to high transverse magnetic fields at cryogenic temperatures exhibit incompressible electronic states characterized by the Integral (I) and Fractional (F) Quantized Hall Effects (QHE), where the Hall resistances are quantized as $R_{xy} = h/(p/q)e^{2}$^1,2^. Here, IQHE are a single particle localization or an absence of backscattering effect with $p/q = 1,2,3\ldots$, while FQHE are many body electronic correlation effects in a magnetic field that are associated with mostly odd-denominator and some even-denominator rational-fractions, *p*/*q*^1–3^. Graphene, a linearly dispersed material^4,5^, has recently become a popular system for studying FQHE^6–12^. However, the GaAs/AlGaAs system, which served to provide some key discoveries^1–3^, due to its extra-ordinarily high quality, is still a material of choice for studying related phenomena. FQHE occur most prominently, for Landau level filling factors $\nu \le 1$, at $\nu = p / (2kp \pm 1)$, and the easily observable FQHE occur here with $k=1$ for the $'+'$ case at $\nu = 1/3, 2/5, 3/7, \ldots$ and for the $'-'$case at $\nu = 1, 2/3, 3/5, 4/7\ldots$, about the one-half filled Landau level^13,14^. In the GaAs/AlGaAs 2D electron system, spin is an extra degree of freedom, and this leads to a double degeneracy of Landau levels that is removed by the Zeeman spin splitting, $g \mu _B B$, which is small compared to the Landau level cyclotron energy splitting, $\hslash \omega _{c}$. Hence, in experiments, it is expected and observed that between $1 \le \nu \le 2$, FQHE become manifested also in the upper spin subband with $k=1$ at $\nu = 1 + p/(2p \pm 1)$. Thus, FQHE could occur, for example, at $\nu = 4/3, 7/5, 10/7, \ldots$ for $\nu \le 3/2$ and at $\nu =2,5/3, 8/5, 11/7\ldots$ for $\nu \ge 3/2$^1–3^. A small Zeeman splitting can also lead to a competition between spin polarized and spin unpolarized many ground body states at a given filling factor^15–19^. Previous studies have reported a symmetry in filling factors that maps $\nu <-> 2- \nu$, which implies that $1/3 <-> 5/3$, $2/5 <-> 8/5$, and $2/3 <-> 4/3$^3,20^. Since 1/3 is spin polarized like the 1 state, and 2/5 or 2/3 could be unpolarized like the 2 state, the symmetry implies that 5/3 is spin polarized and remains so, while the 8/5 and the 4/3 can undergo an unpolarized to partially polarized spin transition with a change in the Zeeman energy relative to the interaction energy^3,20^. More generally, it is believed that even numerator FQHE such as, say, the 4/7 or 4/9 can exhibit a multiplicity of spin polarized states and it is possible to observe transitions between these states by tuning the Zeeman energy relative to the correlation energy. And, odd numerator (p) FQHE are fully spin polarized for p=1 and partially polarized for $p\ge 3$^3,21^. Here, we report and examine a marginal metallic state with a vanishing gap at $\nu = 8/5$ in the untilted GaAs/AlGaAs 2DES specimen, and at $\nu = 4/3$ in a $\theta = 66^{0}$ tilted magnetic field, through a microwave photo-excited transport study of the GaAs/AlGaAs 2DES. The results presented here suggest, remarkably, that at the possible degeneracy point of states with different spin polarization, where the 8/5 or 4/3 FQHE vanish, there occurs a peculiar marginal metallic state that differs qualitatively from a quantum Hall insulating state and the usual quantum Hall metallic state. We show that such a marginal metallic state occurs most prominently in a $n= 2 \times 10^{11} cm^{-2}$ case at 8/5 for the untilted 2DES, and in a $n=1.2 \times 10^{11} cm^{-2}$ case at 4/3 under $\theta = 66^{0}$ tilt as mentioned above, over the interval $1 \le \nu \le 2$, that also includes the $\nu = 3/2$ state, which appears perceptibly gapped in the first instance.

## Results

### Marginal metallic state at $\nu = 8/5$

**Figure 1:**
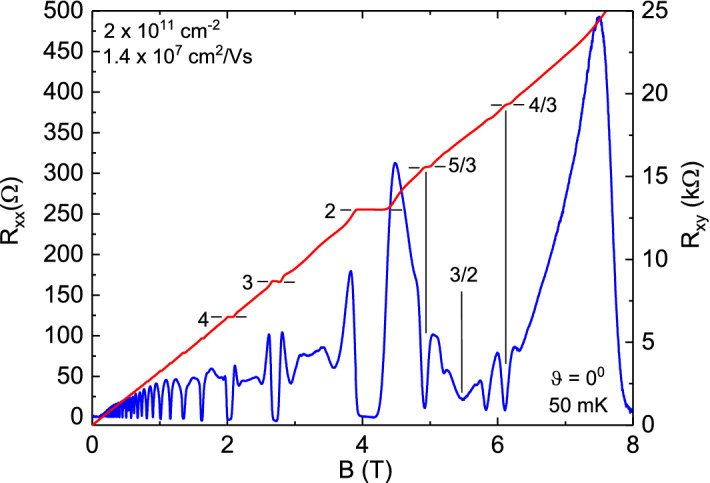
Overview of the diagonal and Hall resistances versus the magnetic field in a GaAs/AlGaAs heterostructure The diagonal resistance $(R_{xx})$ and Hall resistance $(R_{xy})$ are shown with short horizontal line segments marking some Integral (I) and Fractional (F) Quantized Hall Effects (QHE). Vertical lines indicate filling factors for the 4/3 and 5/3 FQHE and the 3/2 state. The carrier density and the mobility are indicated in the top left. The temperature and the tilt angle are indicated on the lower right.

**Figure 2:**
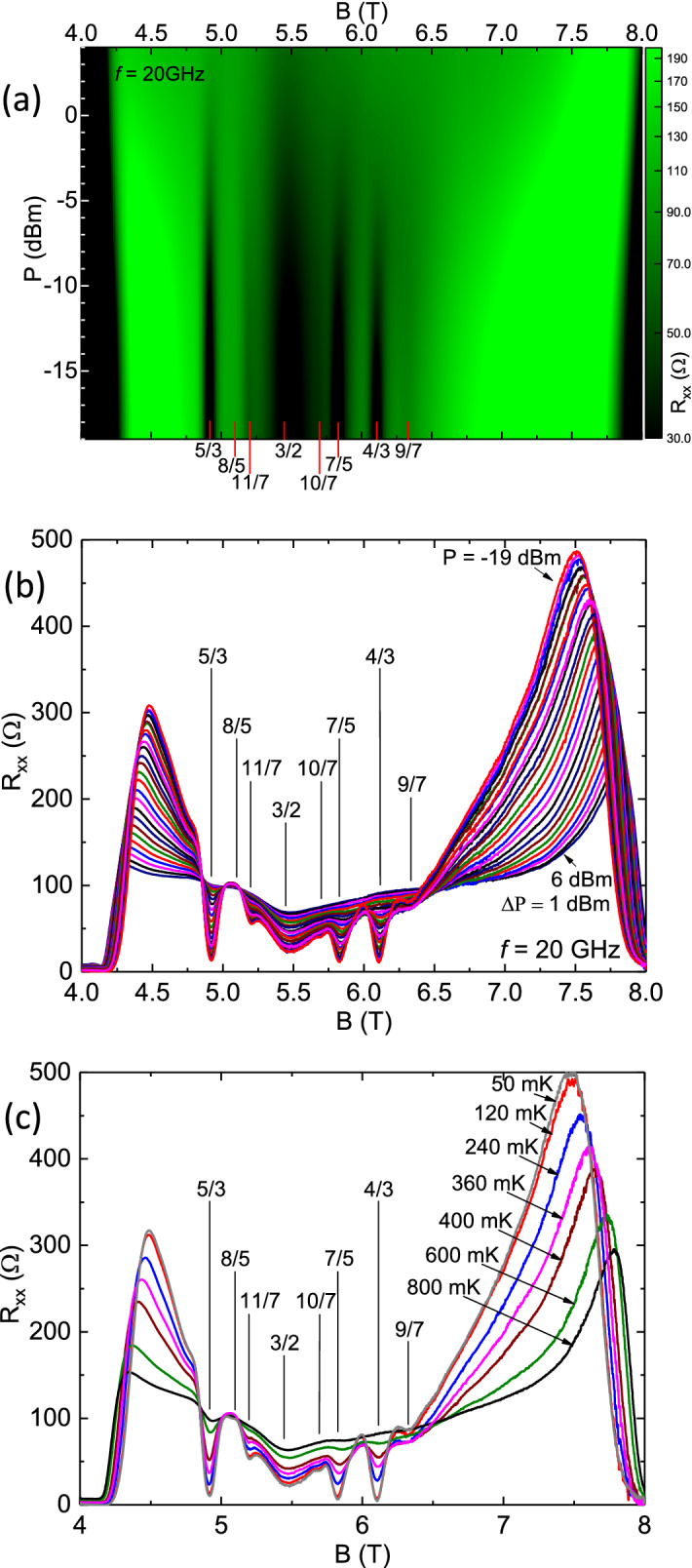
Microwave power ((**a**),(**b**)) and temperature (**c**) evolution of the diagonal resistance $R_{xx}$ between $4 \le B \le 8T$ ($1< \nu < 2$). (**a**) Color plot of $R_{xx}$vs. *B* and vs. *P* at $f=20GHz$. *P* is specified in *dBm* (decibel - milliwatts) (**b**) $R_{xx}$ vs. *B* is plotted at various microwave power *P* between $-19 \le P \le 6$ *dBm* in steps $\Delta P = 1 dBm$. Some filling factors of interest are marked with vertical lines. (**c**) $R_{xx}$ vs. *B* is plotted at temperatures $50 \le T \le 800 mK$. Some filling factors of interest are marked with vertical lines. Note the absence of a resistance minimum or maximum, or temperature sensitivity, at $\nu = 8/5$ in all three panels.

**Figure 3:**
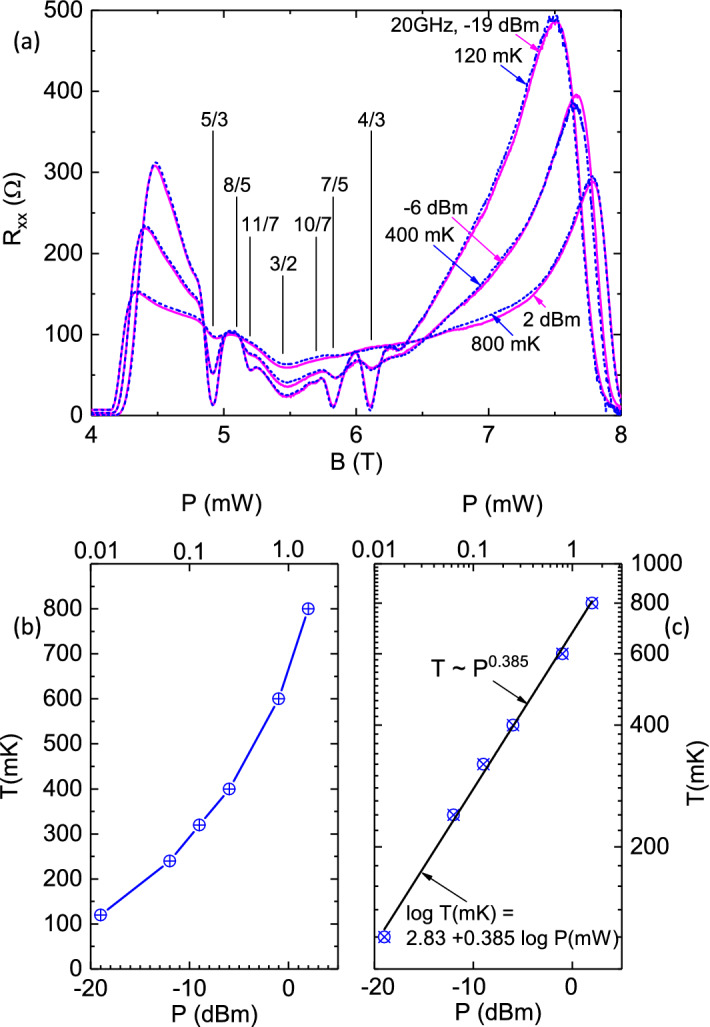
Overlay of power- and temperature- traces of $R_{xx} vs. B$ and the extraction of the carrier temperature at a given microwave power. (**a**) $R_{xx}$ vs. *B* traces obtained at various *P* at base temperature, have been overlaid upon dark $R_{xx}$ vs. *B* traces obtained at some temperatures, *T*. The results suggest excellent registry between the power and temperature traces over the entire exhibited span of magnetic fields $4 \le B \le 8 T$. (**b**) The carrier temperatures (*T*) at different microwave powers (*P*), which have been extracted by matching up power traces with temperature traces, as in the top panel. (**c**) A log-log plot suggests simple power law behavior with $T\approx P^{0.385}$.

**Figure 4:**
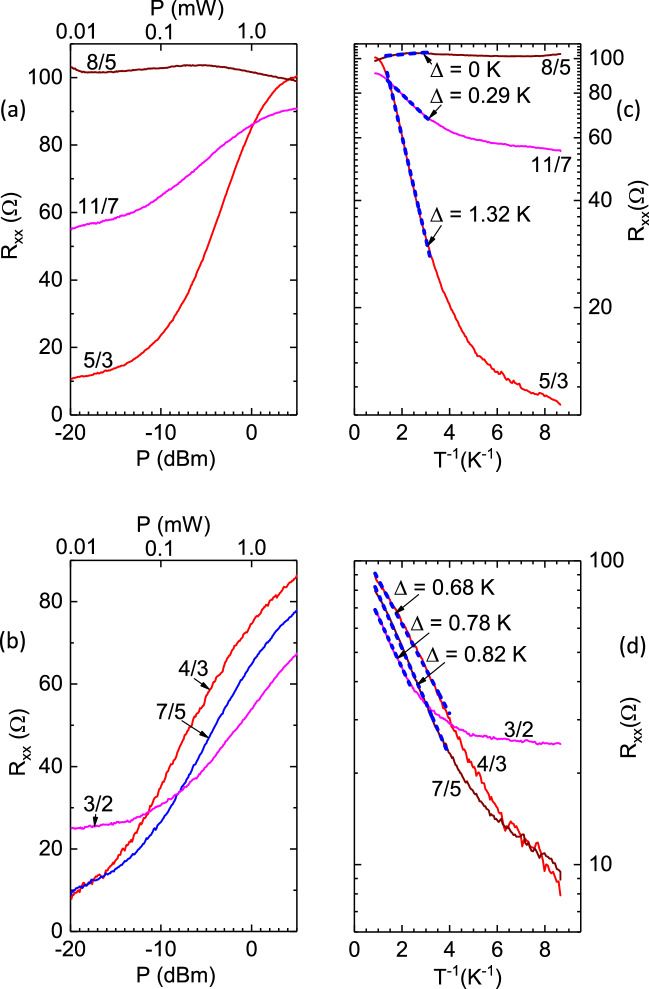
Extraction of activation energy at various filling factors of interest. (**a**,**b**) $R_{xx}$ vs *P* traces obtained with the specimen at base temperature. The panel (**a**) shows the results for filling factors 5/3, 8/5 and 11/7 , while the panel (**b**) shows the same for filling factors 4/3, 7/5, and 3/2. ((c),(d)) For these panels, the abscissa *P* of the left panels have been converted to the the inverse temperature $T^{-1}$ using the calibration exhibited in the Fig. 3c. These panels also exhibit an activation fit, which is indicated by the dashed blue line, for each trace. Note that the activation energy $\Delta$ vanishes only for filling factor 8/5, indicating a gapless metallic state at 8/5. This feature is consistent with the absence of the FQHE resistance minimum at $\nu = 8/5$ in Fig. 2. Note also the finite activation energy, $\Delta (3/2) = 0.78K$, at $\nu = 3/2$.

Figure 1 illustrates the transport characteristics of a GaAs/AlGaAs single heterostructure specimen characterized by $n = 2 \times 10^{11} cm^{-2}$ and $\mu = 1.4 \times 10^{7} cm^{2}/Vs$ cooled to a temperature $T=50mK$ in a dilution refrigerator. Here, the magnetic field *B* is oriented perpendicular to the current *I* and the plane of the 2DES. Hence, the tilt angle $\theta = 0 ^{0}$. The figure shows that the diagonal resistance, $R_{xx}$ exhibits Shubnikov-de Haas (SdH) type oscillatory resistance as the Hall resistance $R_{xy}$ exhibits a linear increase at low magnetic fields, $B \le 1T$. At higher fields, the $R_{xx}$ vanishes as the $R_{xy}$ exhibits prominent IQHE which have been marked for $p/q = 4,3,2$ upto $B= 4.5T$^22–24^. At even higher magnetic fields, $R_{xx}$ minima associated with FQHE become observable, and the most prominent FQHE associated with the $p/q=5/3$ and the $p/q = 4/3$ state have been marked here along with the filling factor $\nu = 3/2$^25^. In order to illustrate the marginal metallic state at $\nu = 8/5$, we examine further the transport characteristics in the magnetic field interval $4 \le B \le 8T$, which corresponds roughly to $1 < \nu \le 2$ in Figs 2, 3 and 4.

A distinguishing feature of our transport study is the use of microwave photo-excitation^26–32^ to change the response of the 2DES, here in the FQHE regime. For this purpose, a microwave coaxial line terminated by a magnetic dipole wire loop that winds around the specimen, was installed into the cryostat to convey externally generated microwaves to the sample. The microwaves were generated with a computer controlled frequency (*f*)- and power (*P*)-tunable- microwave synthesizer, and they were applied to the specimen via the coaxial line while the refrigerator maintained base temperature.

Fig. 2a exhibits a color plot of $R_{xx}$ with *B* on the abscissa, and *P* on the ordinate, at $f = 20GHz$. The $R_{xx}$ color scale is shown on the right of Fig. 2a. Here, the ordinate scale units, *dBm* (decibel-milliwatt), is a logarithmic power scale defined by the relation $dBm = 10 log (P (mW)/1mW)$. The specified power (*P*) levels are determined at the source and do not account for insertion losses, losses due to attenuation in the coaxial line, and other mismatch effects. The $f=20GHz$ corresponds to $T=hf/k_{B} = 0.96K$; it was chosen to be low enough to preclude the possibility of single particle spin-resonance over the investigated magnetic field range. The color plot shows FQHE resistance minima at 5/3, 11/7, 10/7, 7/5, 4/3 and 9/7. There is also a broad minimum around 3/2. Note the absence of a resistance minimum or a dark band at 8/5. At this color scale, roughly speaking, 9/7 minimum vanishes around $-6dBm$, 4/3 disappears around $P= -3dBm$, 7/5 vanishes around 0*dBm*, 11/7 persists to around $+2dBm$ and 5/3 is still observable at +4*dBm*. The relative persistence of a FQHE to higher powers indicates, we believe, a more robust excitation gap. Among the observed FQHE, the 7/5 exhibits the broadest minimum in Fig. 2(a), although it is not the FQHE that persists to the highest *P*. Figure 2(b) exhibits the associated $R_{xx}$ vs. *B* traces at $f= 20 GHz$ with the power level stepped between $-19 \le P \le 6dBm$ at increments of $\Delta P = 1 dBm$. As in Fig. 2(a), for $\nu \le 3/2$, the figure 2(b) shows prominent FQHE resistance minima at 4/3, 7/5,  and weaker minima at 10/7 and 9/7, and, for $\nu \ge 3/2$, at 5/3 and 11/7 . The figure conveys a smooth and progressive change in the resistance traces, which is characterized by a decrease in the amplitude of the oscillatory resistance variation, as the power level is increased towards 6*dBm* or 4*mW*^28^. It is interesting to note that although the $R_{xx}$ minima associated with the 11/7 and the 9/7 appear rather weak in Fig. 2(b), they persist to rather high *P* in the color plot of Fig. 2(a). Indeed, 11/7 persists to higher *P* than the 4/3 or 7/5 in Fig. 2(a). Since the observed resistance variation with increased *P* is reminiscent of the effect of a temperature change^29,30^, transport measurements were also carried out in the dark, i.e., in the absence of microwave excitation, as a function of the temperature and these results are shown in Fig. 2(c) for $50 \le T \le 800 mK$. As is evident in Fig. 2(c), increasing the temperature decreases the amplitude of the oscillatory variation in the same manner that increasing *P* provides for the same function in Fig. 2(b). Again, a feature in all panels of Fig. 2 is the absence of a resistance minimum, a necessary condition for FQHE, at filling factor 8/5. Another feature is the remarkable insensitivity of the $R_{xx}$ on both the microwave power *P* in Fig. 2(b) and on the temperature in Fig. 2(c) at $\nu = 8/5$. Indeed, it looks like $\nu = 8/5$ is one of the few places showing a pronounced power- and temperature- insensitivity in the $R_{xx}$ over the entire *B* span. Another location showing similar *P*- and *T*- insensitivity is the oscillatory node at $B \sim 4.85T$. Note also the concurrent stronger power- and temperature- dependence in Fig. 2(b) and (c), respectively, of the $R_{xx}$ at filling factor 3/2. These results suggest the existence of a marginal metallic state at 8/5 and a gapped state at 3/2 in these specimens in this high mobility, high density condition.

In the high magnetic field limit, $R_{xx} \sim \rho _{xx} = \sigma _{xx}/\sigma _{xy}^{2}$. Thus, an insulating state, which is characterized by a vanishing conductivity $\sigma _{xx} -> 0$ exhibits also a vanishing resistance ($R_{xx} ->0$), while enhanced conductivity corresponding to a metallic state corresponds to enhanced resistance, at lower temperatures. Since an insulating state exhibits vanishing conductivity in the $T-> 0$ limit, $R_{xx}$ minima associated with FQHE are viewed as insulating states. On the other hand, SdH oscillatory maxima, which typically exhibit increasing conductivity with decreasing temperatures, are essentially metallic states. The results exhibited here for the 8/5 state suggest neither a decreasing conductivity with a decreasing temperature as at the usual FQHE resistance minima (insulating state), nor an increasing conductivity with decreasing temperature as at a SdH oscillation maxima (metallic state)^33^. The resistance at 8/5 is mostly temperature- and power- independent. For this reason, the observed characteristics at 8/5 are referred to as a marginal metallic state. Further results shown below confirm this point.

The remarkable similarity between the variation in the $R_{xx}$ vs. *B* traces with *P* in Fig. 2 (b), and with *T* in Fig. 2(c), respectively, motivated an effort to correlate the power- and temperature- effects and extract a possible heating effect due to microwaves on the 2DES in the FQHE regime. The results are exhibited in Fig. 3. Fig. 3(a) shows an overlay of resistance traces obtained at different *P* at base temperature, with different dark traces at elevated temperatures. We have found that a photo-excited trace at any power level at base temperature could be matched with a dark trace obtained at some elevated temperature over the entire exhibited *B*-span. For example, Fig. 3(a) shows that the $P=-19 dBm$ $R_{xx}$ vs.*B* trace at base temperature is identical to the $R_{xx}$ vs. *B* dark trace obtained at 120*mK*. Similarly, the $P=-6dbm$ photo-excited trace matches the $T=400 mK$ dark trace, and the $P=2dBm$ trace can be overlaid on the 800*mK* dark trace. Again, the remarkable feature is that a photo-excited trace obtained over the entire span $4 \le B \le 8T$ at some *P* is nearly identical to a similar dark trace obtained over the same *B* interval at some *T*. Although this interval includes a mix of both FQHE and non-FQHE states, the match is just as good everywhere.

By overlaying and matching the power and temperature dependent $R_{xx}$ vs. *B* traces, we extracted the effective temperature at each *P* level; the extracted temperature *T* is plotted vs. *P* in Fig. 3 (b). Here, the *T* appears to increases superlinearly with the *P*, with *P* specified in the logarithmic *dBm* scale. A log-log plot of the *T* vs. *P* , see Fig. 3(c), indicates a straight line relationship that suggests $T(mK) \sim ( P(mW))^{0.385}$. Below, this “calibration” will serve to convert *P* to *T*, when the specimen is photo-excited at $f=20 GHz$ at base temperature. We note, parenthetically, that since microwave power $P \sim E^{2}$ where E is the amplitude of the microwave electric field, $T \sim P^{0.5}$ could imply that the carrier temperature increase is proportional to the microwave electric field. Of course, the exponent observed here is not quite 0.5^31,32^.

Fractional quantum Hall states are characterised by gap energies for quasiparticle-quasihole excitations and these gaps are typically measured from the temperature dependence of the FQHE $R_{xx}$ minima in the thermally activated regime^1^. As we show in the following, we can determine these gaps by measuring the microwave power variation of the $R_{xx}$ minima with the specimen at base temperature, followed by a conversion of *P* to *T* using the “calibration” mentioned above. We note, that from the experimental point of view, there are some definite experimental advantages to such microwave based measurements over the conventional temperature dependent measurements: i) The applied microwave power can be controlled with great precision at the microwave source and, therefore, very small incremental changes in temperature appear possible at the sample with small power changes at the source, ii) The source microwave power (and therefore, in principle, the temperature) can be varied smoothly at the desired rate. iii) Since microwave induced heating occurs very locally at the specimen and the heated volume depends on the size of the magnetic dipole wire loop around the specimen, rapid heating and cooling with small time constants can be realized very easily. vi) Feedback loops utilized in typical temperature controlled studies in dilution refrigerators, which can lead to temperature oscillations, do not occur here. v) Possible magnetic field sensitivity of thermometers utilized in feedback loops, which could lead to spurious temperature changes, when one tries to control to a fixed sensor value, also do not occur here. More simply, one might say that it is a lot easier to measure, change, and control the microwave power than it is to change, actively control, and measure the temperature inside a dilution refrigerator. The principal requirement for the utility of such measurements using microwave sources seems to be the long term stability of the base temperature in the dilution refrigerator system. We have had not much trouble maintaining this requirement for such experimental runs.

Figure 4 (a) shows measurements of $R_{xx}$ vs. *P* at $\nu = 5/3,8/5$, and 11/7 for $\nu > 3/2$, while Fig. 4(b) shows the same for $\nu = 4/3, 7/5$, and 3/2. In Fig. 4(a), a strong increase of $R_{xx}$ vs. *P* is indicated for $\nu = 5/3, 11/7$, which implies a power and, therefore, a temperature sensitive $R_{xx}$, while at $\nu = 8/5$, the $R_{xx}$ is insensitive to *P* and therefore also to *T*. On the other hand, at $\nu = 4/3, 7/5, 3/2$, the $R_{xx}$ shows a strong increase with *P* and the variation at these $\nu$ is similar, especially at higher *P*. These features in both Fig. 4(a) and Fig. 4(b) are fully consistent with the data exhibited in Fig. 2(b), and they confirm the notion of a marginal metallic state at 8/5. Since FQHE states are characterized by gap energies for quasiparticle-quasihole excitations, which are usually determined from activation energies extracted from *LogR* vs $T^{-1}$ plots, we replotted the $R_{xx}$ vs. *P* traces of Fig. 4(a) and Fig. 4(b) as (log-scale) $R_{xx}$ vs. $T^{-1}$ , in Fig. 4(c) and Fig. 4(d), respectively, using the power-temperature “calibration” exhibited in Fig. 3(c). Fig. 4(c) and (d) indicate linear variation, which is identified by the blue dashed lines in the large temperature (small $T^{-1}$) limit. A feature in Fig. 4(c) is that, $\Delta (5/3)> \Delta (11/7) > \Delta (8/5)$. Indeed, $\Delta (8/5) = 0$ within experimental uncertainties, confirming a gapless ($\Delta = 0$), marginal metallic state at $\nu = 8/5$. Another feature, this one observable in Fig. 4(d), is that the high temperature (small $T^{-1}$) slopes for $\nu = 4/3, 7/5,$ and 3/2 are similar, with $\Delta (7/5)> \Delta (3/2) > \Delta (4/3)$, and remarkably, there appears to be an activation gap at 3/2. From the data of Fig. 2(b), it is apparent that the nearly uniform temperature variation in $R_{xx}$ between $3/2 \ge \nu \ge 4/3$, especially at higher *P*, is the reason for the similar $\Delta$ over this range, as indicated in Fig. 4(d).

### Marginal metallic state at $\nu = 4/3$ in a $\theta = 66^{0}$ tilted 2DES

**Figure 5:**
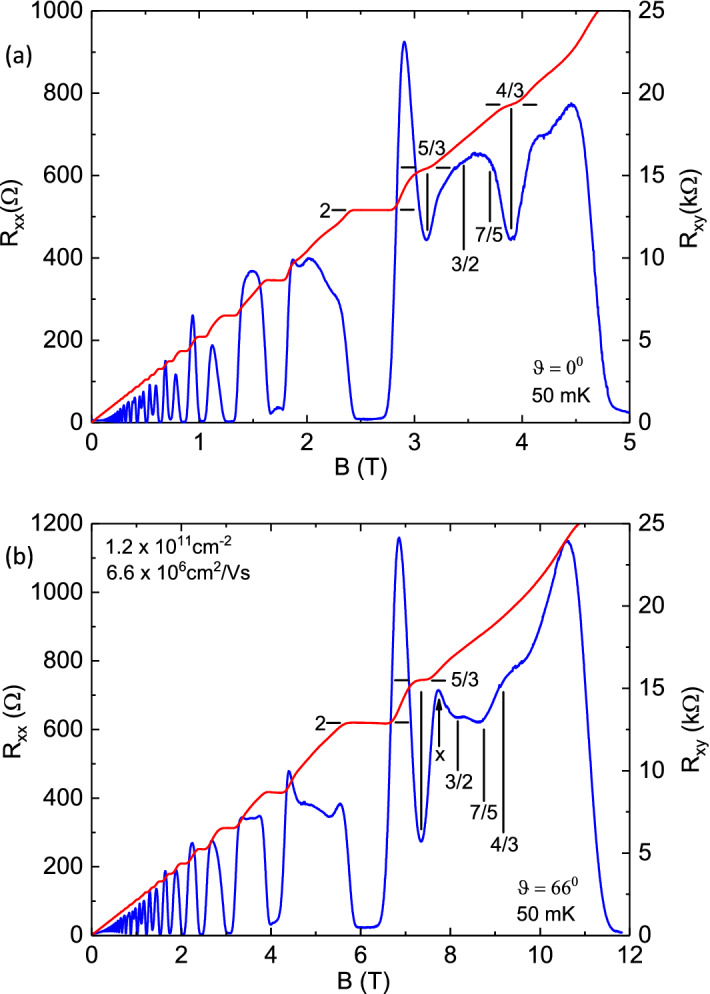
The diagonal and Hall resistances versus the magnetic field in a GaAs/AlGaAs heterostructure in the low mobility, low density condition. The diagonal resistance $(R_{xx})$ and Hall resistance $(R_{xy})$ are shown with some marked Integral (I) and Fractional (F) Quantized Hall Effects (QHE). (**a**) The $R_{xx}$- and $R_{xy}$- vs *B* at a tilt angle $\theta = 0^{0}$, where the sample normal is parallel to the magnetic field, i.e., $\theta = 0^{0}$. (**b**) The results at a tilt angle $\theta = 66^{0}$.

The GaA/AlGaAs heterostructure devices examined for the study above exhibited also a low density ($n \sim 1.2 \times 10^{11} cm^{-2}$), low mobility ($\mu \sim 6.6 \times 10^{6} cm^{2}/Vs$) condition, when the specimens were cooled in the dark^34^. These specimens, in this low mobility condition, exhibited fewer perceptible FQHE over the interval $1 \le \nu \le 2$. Yet, the 5/3 and 4/3 FQHE’s were prominent in the untilted ($\theta = 0^{0}$) condition, with the specimen normal parallel to the magnetic field, see Fig. 5(a). We tilted the specimen with respect to the magnetic field in order to extinguish the 4/3 FQHE state and found a similar marginal metallic state, at a tilt angle of $\theta = 66^{0}$ of the specimen normal with respect to the magnetic field, see Fig. 5(b). In comparing Fig. 5(a) and Fig. 5(b), we observe that the $\nu = 4/3$ resistance minimum of Fig. 5(a) becomes unobservable in Fig. 5(b). An imperceptible $\nu =7/5$ resistance minimum in Fig. 5(a) becomes observable in Fig. 5(b). There is also a resistance peak on the high field side of 5/3 in Fig. 5(b), marked as “x” at $B \sim 7.75T$, which is imperceptible in Fig. 5(a). Below, we report further details of the study in this tilted field situation.

**Figure 6:**
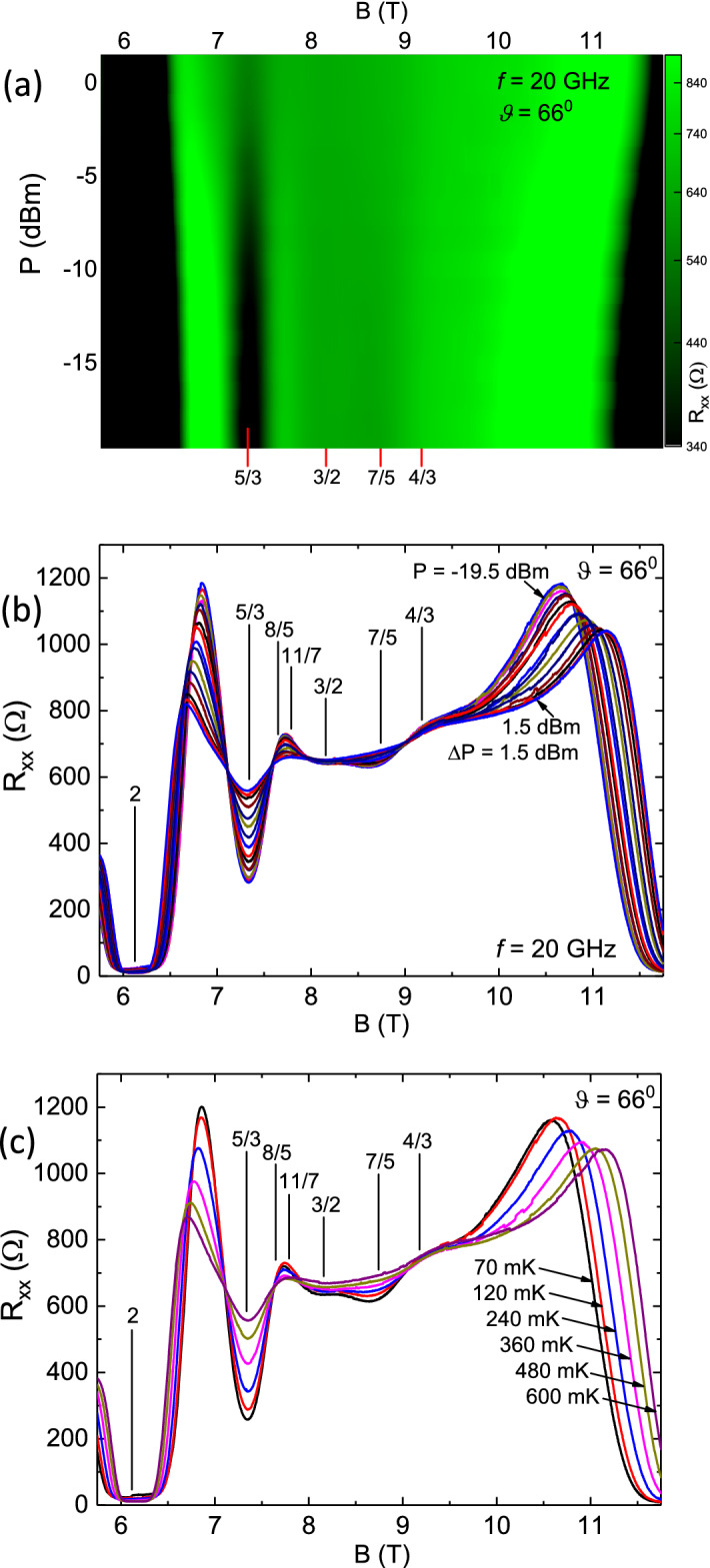
Microwave power (**a**,**b**) and temperature (**c**) evolution of the diagonal resistance $R_{xx}$ between $5.75 \le B \le 11.75T$ at a tilt angle $\theta = 66^{0}$. (a) Color plot of $R_{xx}$vs. *B* and vs. *P* at $f=20GHz$. *P* is specified in *dBm* (decibel - milliwatts) (b) $R_{xx}$ vs. *B* is plotted at various microwave power *P* between $-19.5 \le P \le 1.5$ *dBm* in steps $\Delta P = 1.5 dBm$. Some filling factors of interest are marked with vertical lines. (c) $R_{xx}$ vs. *B* is plotted at temperatures $70 \le T \le 600 mK$. Some filling factors of interest are marked with vertical lines. Note the absence of a resistance minimum or maximum, or temperature sensitivity, at $\nu = 4/3$ in all three panels.

Fig. 6(a) exhibits a color plot of $R_{xx}$ with *B* on the abscissa, and *P* on the ordinate, at $f = 20GHz$. The $R_{xx}$ color scale is shown on the right of Fig. 6(a). The color plot shows a strong FQHE resistance minimum about 5/3. Note the absence of a resistance minimum or a dark band at 4/3 in this case. Note also that the 5/3 appears to persist to $P \ge 2 dBm$. Figure 6(b) exhibits the associated $R_{xx}$ vs. *B* traces at $f= 20 GHz$ with the power level stepped between $-19.5 \le P \le 1.5dBm$ at increments of $\Delta P = 1.5 dBm$. As in Fig. 6(a), for $\nu \le 3/2$, the figure 6(b) shows a prominent FQHE resistance minima at 5/3. An interesting feature is the broad resistance maximum between 8/5 and 11/7, also marked as “x” in Fig. 5(b), although there are apparently no resistance minima visible near the 8/5 and 11/7 filling factors. There is also a $R_{xx}$ minimum in the vicinity of 7/5 at this tilt angle although the 7/5 resistance minimum is not apparent in the untilted specimen, see Fig. 5(a). The figure 6(b) conveys a smooth and progressive change in the resistance traces, which is characterized by a decrease in the amplitude of the oscillatory resistance variation, as the power level is increased towards 1.5*dBm*. Once again, transport measurements were also carried out in the dark, i.e., the absence of microwave excitation, as a function of the temperature and these results are shown in Fig. 6(c) for $70 \le T \le 600 mK$. As is evident in Fig. 6(c), increasing the temperature decreases the amplitude of the oscillatory variation, just as increasing *P* provides for the same function in Fig. 6(b). Again, a feature in all panels of Fig. 6 is the absence of a resistance minimum, at filling factor 4/3. Another feature is the remarkable insensitivity of the $R_{xx}$ on both the microwave power *P* in Fig. 6(b) and on the temperature in Fig. 6(c) at $\nu = 4/3$. These results suggest the existence of a marginal metallic state also at 4/3 in a tilted field configuration.

**Figure 7:**
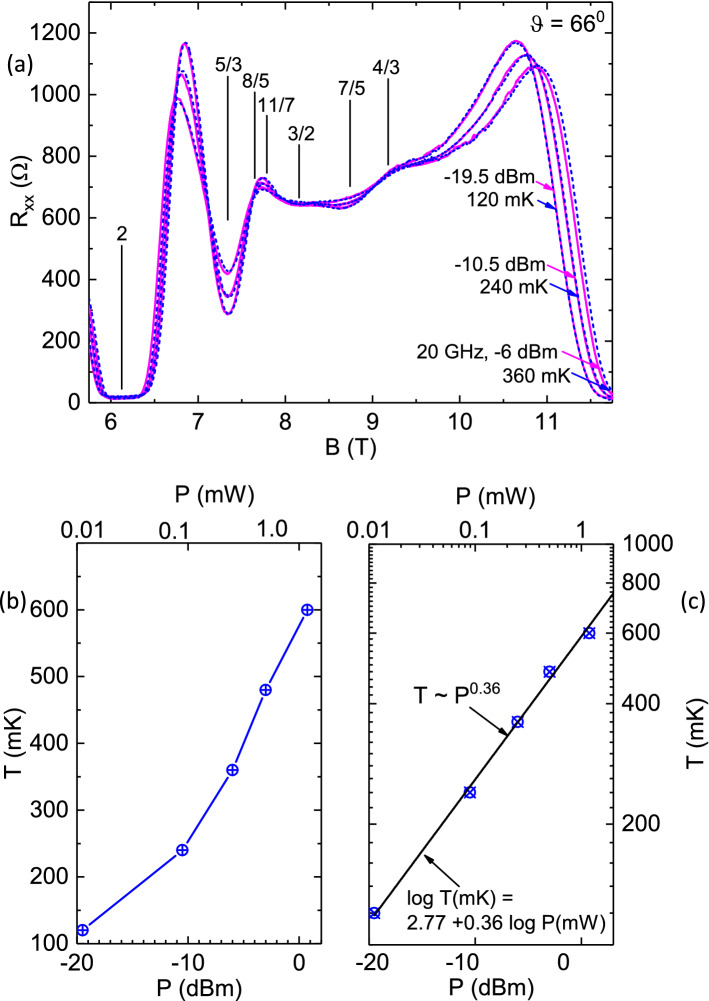
Overlay of power- and temperature- traces of $R_{xx} vs. B$ and the extraction of the carrier temperature at a given microwave power. (**a**) $R_{xx}$ vs. *B* traces obtained at various *P* at base temperature, have been overlaid upon the dark $R_{xx}$ vs. *B* traces obtained at some temperatures, *T*. The results suggest excellent registry between the power and temperature traces. (**b**) The carrier temperature (*T*) at different microwave power (*P*), which has been extracted by matching up power traces with temperature traces, as in the top panel. (**c**) A log-log plot suggests simple power law behavior with $T\approx P^{0.36}$.

The similarity between the variation in the $R_{xx}$ vs. *B* traces with *P* in Fig. 6 (b), and with *T* in Fig. 6(c), respectively, served to extract the heating effect due to microwaves on the 2DES. Fig. 7(a) shows an overlay of photo-excited resistance traces obtained at different *P* at base temperature, with different dark traces at elevated temperatures. Fig. 7(a) shows that the $P=-19.5 dBm$ $R_{xx}$ vs.*B* trace at base temperature is identical to the $R_{xx}$ vs. *B* dark trace obtained at 120*mK*. Similarly, the $P=-10.5dbm$ photo-excited trace matches the $T=240 mK$ dark trace, and the $P=-6dBm$ trace can be overlaid on the 360*mK* dark trace. Thus, photo-excited trace obtained over $5.75 \le B \le 11.75T$ at some *P* matches a similar dark trace obtained over the same *B* interval at some elevated *T*. By matching the power and temperature dependent $R_{xx}$ vs. *B* traces, we extracted the effective temperature at each *P* level; the extracted temperature *T* is plotted vs. *P* in Fig. 7 (b). A log-log plot of the *T* vs. *P* , see Fig. 7(c), indicates that $T(mK) \sim ( P(mW))^{0.36}$, which is in agreement, within experimental uncertainties, with the results shown in Fig. 3(c). This “calibration” for $\theta = 66^{0}$ will serve to convert *P* to *T*, when the specimen is photo-excited at $f=20 GHz$ at base temperature, at this tilt angle.

**Figure 8:**
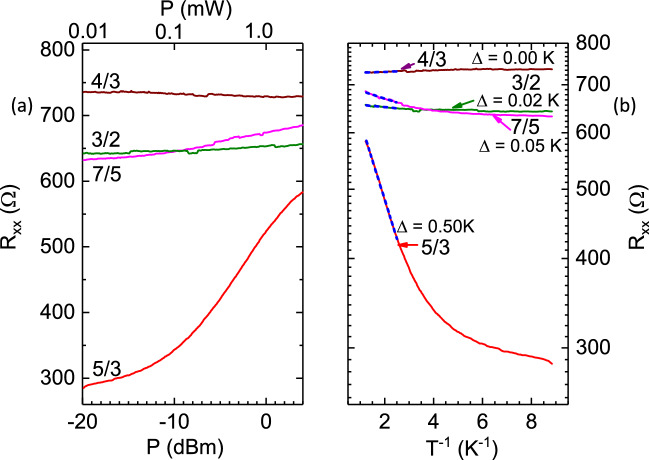
Extraction of activation energy. (**a**) $R_{xx}$ vs *P* traces obtained at the indicated filling factors with the chip carrier held at base temperature. The panel (**a**) shows the results for filling factors $\nu = 5/3$, 4/3, 7/5, and 3/2. (**b**)Here, the abscissa *P* of panel (**a**) has been converted to the the inverse temperature $T^{-1}$ using the calibration exhibited in the Fig. 7(c). An activation fit, which is indicated by the dashed blue line, is shown for each trace. Note that the activation energy $\Delta$ vanishes for filling factor 4/3, indicating a gapless metallic state at 4/3 at a tilt angle $\theta = 66^{0}$. This feature is consistent with the absence of the FQHE resistance minimum at $\nu = 4/3$ in Fig. 6 (b) and (c).

Figure 8 (a) shows measurements of $R_{xx}$ vs. *P* at $\nu = 5/3,4/3, 7/5$, and 3/2. In Fig. 8(a), a strong increase of $R_{xx}$ vs. *P* is indicated for $\nu = 5/3$, which implies a strongly temperature dependent $R_{xx}$ at this filling factor. The $R_{xx}$ at 7/5 and 3/2 show subdued increase with *P*, while the $R_{xx}$ at 4/3 is virtually insensitive to *P*. To extract activation energies, we replotted the $R_{xx}$ vs. *P* traces of Fig. 8(a) as (log-scale) $R_{xx}$ vs. $T^{-1}$ , in Fig. 8(b), using the power-temperature “calibration” exhibited in Fig. 7(c). Fig. 8(b) indicates linear variation in the large temperature (small $T^{-1}$) limit, which is identified by the blue dashed lines in Fig. 8(b). Fig. 8(b) shows that, $\Delta (5/3)> \Delta (7/5) > \Delta (4/3)$. Indeed, $\Delta (4/3) = 0$ within experimental uncertainties, confirming a gapless ($\Delta = 0$), marginal metallic state at $\nu = 4/3$ in this instance.

## Discussion

FQHE’s with even numerators, and odd numerators greater than one, can, in principle, include a multiplicity of spin polarized states for the same FQHE^3,20^. Crossovers can occur between these different polarization states as a function of a change in the Zeeman energy relative to the interaction energy. The literature indicates that a FQHE minima can disappear when two different spin polarization states become degenerate in energy^16–20,35,37–41^. We have studied the absence of FQHE at 8/5 at $\theta = 0^{0}$ and $n=2 \times 10^{11} cm^{-2}$, and at 4/3 at $\theta = 66^{0}$ and $n=1.2 \times 10^{11} cm^{-2}$ , which is presumably induced by the coming together in energy of different polarization states associated with the 8/5 and 4/3 FQHE, respectively. The question of interest for this study was: what is the character of the electrical response in such a case? Is the response similar to that of a QHE insulator ($\sigma _{xx} -> 0$ at $T-> 0$) or a QHE metal, i.e., like the peaks of SdH oscillations of electrons or composite fermions, where $\sigma _{xx}$ increases with decreasing temperatures.

We reported here that near the degeneracy of different spin polarization states of the 8/5 or 4/3 FQHE’s, there is a marginal metallic state that is qualitatively different from the QHE insulating state and the usual high field metallic state observed at the peaks of SdH oscillations of electrons or composite fermions in the sense that, here, the $R_{xx}$ or $\sigma _{xx}$ is essentially insensitive to temperature and microwave power. The term “marginal metallic state” conveys the idea that the conductivity neither rapidly drops to zero with decreasing temperatures, nor does it increase greatly with decreasing temperatures. These marginal metallic states at specific odd-denominator rational fractional filling factors are superficially reminiscent of nodes in the (SdH type) oscillatory resistance: For example, we pointed out that a *P*- and *T*- insensitive point, i.e., a node, in the oscillatory $R_{xx}$ occurs also at $B \sim 4.85T$ in Fig. 2(b) or Fig. 2(c). There also occur *P*- and *T*- insensitive points, i.e., nodes in the oscillatory $R_{xx}$, at, for example, $B \sim 7.11T$ and at $B \sim 7.59T$, in Fig. 6(b) and Fig. 6(c). The more appropriate analogy here, we believe, involves relating these FQHE marginal metallic states with nodes in a beat pattern arising from the interference between different oscillatory frequencies or harmonic components, as in the case of “zero-field spin splitting,”^42,43^ because resistance at the nodes in such a beat pattern would also exhibit temperature and power insensitivity, for a temperature insensitive beat frequency. A possible interpretation then could be that the density or tilt angle influences and modifes the frequencies underlying a beat pattern in this FQHE regime, which shifts the location of the node in the beat, depending upon the difference frequency. Perhaps, such an effect produces the marginal metallic state at the observed odd denominator rational fractional filling factors, where FQHE are normally expected.

It is also interesting to note that although the measured activation energies can be on the order of $\Delta \sim 1K$, see Fig. 4(c),(d), (and the $\nu =5/3$ case in Fig. 8(b)), which is approximately the same as the microwave photon energy $hf/k_{B} = 0.96K$, there was no obvious resonant response to be observed here, at least so far, in the photo-excited traces. Indeed, the striking feature was the observable in Fig. 3(a) and Fig. 7(a), that photo-excited traces obtained at base temperature could be overlaid and matched to a dark trace at an elevated temperature, over the entire examined field interval. Indeed, these experiments indicate that microwave photo-excited transport could constitute a new approach to examining physical phenomena in the FQHE regime.

## Methods

The GaAs/AlGaAs heterostructures used in these studies were characterized by a sheet electron density $n_{0} (1.5K) = 1.2 \times 10^{11} cm^{-2}$ and an electron mobility $\mu (1.5K) = 6.6 \times 10^{6} cm^2/Vs$ upon cooling the specimens in the dark, and $n_{0} (1.5K) = 2 \times 10^{11} cm^{-2}$ and an electron mobility $\mu (1.5K) = 1.4 \times 10^{7} cm^2/Vs$ upon illuminating the specimen with a red LED during cooldown^34^. Hall bars^22–24,44^ were fabricated from this material using standard photolithography. The device length-to-width ratio was $L/W = 1$, and the width $W = 200 \mu m$. Electrical contacts were formed by depositing and alloying Au-Ge/Ni at the Hall bar contact pads. The sample was wired into a chip carrier, loaded into a dilution refrigerator system, and the electrical response was measured using low frequency lock-in based techniques. The sample could be tilted in-situ. Microwaves were conveyed to the specimen using a coaxial cable, which was terminated with a magnetic dipole wire-loop around the specimen. Microwaves were provided from a source which could be tuned under computer control for both the frequency and the power. For the power dependent traces (Fig. 2 (a),(b) and Fig. 6(a), (b)), magnetic field sweeps were carried out at the indicated power levels, with the dilution refrigerator at base temperature. For the temperature dependent traces (Fig. 2(c) and Fig. 6(c)), temperature control was acheived at the specified temperatures, and magnetic fields sweeps were carried out without microwave excitation. For the $R_{xx}$ vs *P* sweeps at fixed *B*, the *B* field was held constant at the specified filling factors at base temperatures, and $R_{xx}$ was collected as *P* was swept with the radiation frequency at $f = 20 GHz$. Similar results were obtained at a number of frequencies in the interval $20 \le f \le 50GHz$. As the microwave attenuation due to the coaxial line increased with *f*, the effective power realized at the specimen decreased with increasing *f*, for a fixed source *P* setting. The observed experimental photoexcited transport results at different *f* could be understood, at least so far, simply as a consequence of this frequency dependent attenuation of the microwave coaxial cable.
